# Multipotent mesenchymal stromal cells enhance insulin secretion from human islets via N-cadherin interaction and prolong function of transplanted encapsulated islets in mice

**DOI:** 10.1186/s13287-017-0646-7

**Published:** 2017-09-29

**Authors:** Elisa Montanari, Raphael P. H. Meier, Redouan Mahou, Jörg D. Seebach, Christine Wandrey, Sandrine Gerber-Lemaire, Leo H. Buhler, Carmen Gonelle-Gispert

**Affiliations:** 1 Department of Surgery, Geneva University Hospitals and Medical Faculty, 1211 Geneva, Switzerland; 2 Institute for Biomaterials and Biomedical Engineering, University of Toronto, Toronto, ON Canada; 3 Division of Immunology and Allergy, Geneva University Hospitals and Medical Faculty, 1211 Geneva, Switzerland; 4 Institute of Chemical Sciences and Engineering, Ecole Polytechnique Fédérale de Lausanne, 1015 Lausanne, Switzerland

**Keywords:** Mesenchymal stromal cells, Human islets, N-cadherin, Cell interaction, Encapsulation

## Abstract

**Background:**

Multipotent mesenchymal stromal cells (MSC) enhance viability and function of islets of Langerhans. We aimed to examine the interactions between human MSC and human islets of Langerhans that influence the function of islets.

**Methods:**

Human MSC and human islets (or pseudoislets, obtained after digestion and reaggregation of islet cells) were cocultured with or without cellular contact and glucose-stimulated insulin secretion assays were performed to assess cell function. The expression of several adhesion molecules, notably ICAM-1 and N-cadherin on islets and MSC, was investigated by qPCR. The role of N-cadherin was analyzed by adding an anti-N-cadherin antibody in islets cultured with or without MSC for 24 h followed by insulin measurements in static incubation assays. Islets and MSC were coencapsulated in new hydrogel microspheres composed of calcium alginate and covalently crosslinked polyethylene glycol. Encapsulated cells were transplanted intraperitoneally in streptozotocin-induced diabetic mice and glycemia was monitored. Islet function was evaluated by the intraperitoneal glucose tolerance test.

**Results:**

In vitro, free islets and pseudoislets cocultured in contact with MSC showed a significantly increased insulin secretion when compared to islets or pseudoislets cultured alone or cocultured without cell-to-cell contact with MSC (*p* < 0.05). The expression of ICAM-1 and N-cadherin was present on islets and MSC. Blocking N-cadherin prevented the enhanced insulin secretion by islets cultured in contact with MSC whereas it did not affect insulin secretion by islets cultured alone. Upon transplantation in diabetic mice, islets microencapsulated together with MSC showed significantly prolonged normoglycemia when compared with islets alone (median 69 and 39 days, respectively, *p* < 0.01). The intraperitoneal glucose tolerance test revealed an improved glycemic response in mice treated with islets microencapsulated together with MSC compared to mice transplanted with islets alone (*p* < 0.001).

**Conclusions:**

MSC improve survival and function of islets of Langerhans by cell-to-cell contact mediated by the adhesion molecule N-cadherin.

**Electronic supplementary material:**

The online version of this article (doi:10.1186/s13287-017-0646-7) contains supplementary material, which is available to authorized users.

## Background

Multipotent mesenchymal stromal cells (MSC) present immunomodulatory properties [1–3], thereby reducing the cell-mediated immune response [4]. MSC secrete bioactive molecules that improve tissue regeneration by increasing vascularization [5–9], angiogenesis [10] and reducing apoptosis [5]. Cotransplantation of islets and MSC results in improved islet graft survival and function as demonstrated by syngeneic and allogeneic transplantation models in rodents and non-human primates [4–6, 9, 11], in a rat-to-mouse xenotransplantation model [3, 7], and in a human to diabetic humanized NOD *scid* gamma mouse model [12]. However, the functionality of human islets cotransplanted with human MSC into immunocompetent diabetic mice has, to our knowledge, not so far been investigated. In a setting of human to mouse transplantation, immune rejection is massive and needs to be overcome by immunosuppressive treatments. As a strategy to avoid such treatments, semipermeable microcapsules can be used to protect cells from the host immune reaction [13, 14]. Semipermeable microcapsules allow the exchanges of nutrients, oxygen, and small molecules (lower than 100 kDa) necessary for maintaining viability of cells [15]. Hence, for the human to mouse islet transplantation we used newly developed biomaterials enabling ionotropic interaction between alginate (Alg) molecules and covalent crosslinking between poly(ethylene glycol) (PEG) derivatives, which have higher mechanical resistance compared to Alg [16].

Further, the molecular mechanism leading to improved islet graft survival and function is unclear. Several studies attributed the beneficial effect to factors released by MSC [17] and also to regulatory effects on the host immune system [4]. It is not known whether direct cell contact between islets and MSC plays a role in the beneficial effects of MSC on islet function. However, adhesion molecules such as cadherins and integrins are expressed in human islets [18, 19] and play a role in regulating insulin secretion. Notably, cadherin interactions on beta cells play a role in increased insulin secretion after glucose stimulation [20] and are implicated in protecting islets from apoptosis [21]. Morphological analysis of human islets demonstrated dispersed alpha and beta cells [22], and only recently structures of subislets have been identified, where alpha cells are organized around centrally located beta cells [23]. This cell arrangement, based on cell interactions between alpha cells and beta cells, and also with stromal cells around islets, is important for an optimal insulin response by beta cells [24]. Further, this particular arrangement is not present in so-called pseudoislets, which are islet cell aggregations obtained in vitro after enzymatic islet dissociation, but reappears after transplantation in mice [25]. The mechanism leading to this rearrangement remains unclear, but likely involves exogenous factors derived from the host environment.

Hence, in this study we analyzed the effect of MSC on islet function as well as their effect on morphology and function of pseudoislets, implying an increased cell contact between MSC and alpha and beta cells. We observed an increased insulin secretion from islets in contact with human MSC in vitro, which prompted us to investigate a possible involvement of intercellular adhesion molecules, such as epithelial (E)-cadherin, neural cell adhesion molecule (NCAM), epithelial (Ep)CAM-1, vascular (V)CAM-1, N-cadherin, and intercellular (I)CAM-1. Furthermore, we aimed to assess the function of human islets coencapsulated with MSC upon transplantation in diabetic mice.

## Methods

### Isolation and culture of human pancreatic islets and human MSC

Human islets were isolated following the Ricordi protocol [26], and their purity was assessed after dithizone staining and calculated using Metamorph (Universal Imaging, West Chester, PA, USA). Islets used for these experiments were 80–100% pure, and no handpicking was performed. Human islets were provided for research only when considered not suitable for clinical transplantation, through the JDRF award 1-RSC-2014-100-1-X, ECIT Islet for Basic Research program. The amount of islets was expressed in islet equivalents (IEQ), normalizing each islet to an average diameter of 150 μm. We considered that 1 IEQ contains 10^3^ cells. Islets were cultured in HEPES-buffered CMRL1066 medium supplemented with 5.6 mmol/L glucose (Gibco-Invitrogen, Basel, Switzerland), 100 IU/ml penicillin, 100 mg/ml streptomycin (P-S; Gibco-Invitrogen), and 10% FCS (Gibco-Invitrogen) (complete CMRL) at 37 °C in humidified air containing 5% CO_2_.

MSC were obtained from the femoral head of patients undergoing total hip replacement as described previously and characterized by FACS analysis and their ability to differentiate into osteoblasts, chondrocytes, and adipocytes [27]. Informed consent was given by all patients and the experimental procedure was approved by the local ethical committee of the University Hospitals of Geneva (NAC 01-015). Briefly, MSC were purified from crushed bone marrow by gradient centrifugation, and then cultured in Iscove’s modified Dulbecco’s medium (Cambrex, Verviers, Belgium) supplemented with 10% FCS, P-S, and 10 ng/ml platelet-derived growth factor BB (PDGF-BB; PeproTech EC Ltd, London, UK) [28]. MSC from three different donors between passages 2 and 4 were used and were cultured at 37 °C in humidified air containing 5% CO_2_.

### Preparation and culture of pseudoislets

Single islet cell suspensions and pseudoislets were prepared as described previously [23, 25]. Briefly, islets were rinsed in PBS (Gibco-Invitrogen) without calcium and incubated for 7 min in Accutase cell detachment solution (Sigma, St Louis, MO, USA) with occasional mixing by pipetting. The resultant single cell suspension was diluted with CMRL supplemented with 10% FCS to stop the enzyme activity. Dissociated islet cells were counted and were cultured overnight at a density of 5 × 10^5^ cells in nonadherent 10-cm diameter Petri dishes in complete CMRL. To obtain pseudoislets, single islet cells (10^4^ cells), with or without MSC at a ratio of 3:1, were taken in 40 μl of complete CMRL or MSC-conditioned medium and placed as microdroplets in the Petri cover, which was inverted to allow cell reaggregation and formation of pseudoislets in the hanging microdroplets. After 3 days, microdroplet medium was renewed and after 6 days microdroplets were collected. Cell clusters recovered from 10 microdroplets (10^5^ islet cells) per well, alone or with MSC, were used for the insulin secretion assay. To obtain MSC-conditioned medium, MSC were cultured in 75 cm^2^ flasks at 80% confluence in complete CMRL and medium was recovered after 48 h. Experiments were performed in triplicate in a 24-well plate. Histology was performed after a 6-day culture.

### Insulin secretion assays

Insulin secretion assay was performed as described previously [29]. Briefly, cells were incubated subsequently for 1 h at 37 °C in basal condition (2.8 mmol/L glucose), stimulated condition (16.7 mmol/L glucose), and stimulated condition containing additionally 5 mmol/L theophylline, to induce maximal insulin secretion. At the end of the assay, the total amount of insulin was extracted with acidic ethanol solution (0.18 mol/L HCl in 70% ethanol). Islets alone (150 IEQ), islets cocultured with MSC (15,000 cells) in cell–cell contact, and islets cocultured with MSC in permeable transwell plates were seeded (cell ratio 10:1) in triplicate in 24-well plates for 3 days in complete CMRL. For blocking experiments, antibodies (25 μg/ml) were added 24 h before performing the secretion assay: either low-endotoxin-azide-free (LEAF) purified anti-human CD325 (N-cadherin), ultra-LEAF purified mouse IgG1κ isotype control (Biolegend, San Diego, CA, USA), or anti-human ICAM-1 antibody (CD54) (R&D Systems, Abingdon, UK). Secreted insulin was measured by ELISA (Mercodia, Uppsala, Sweden), following the manufacturer’s instructions. Values were normalized to the total amount of insulin measured, which was obtained after total cell lysis with acid ethanol. The values of insulin secretion were expressed as the fold increase where the basal condition was set as 1. All experiments were performed in triplicate.

### Real-time RT-PCR

Gene expression of adhesion molecules was analyzed by real-time reverse transcription PCR (RT-PCR), as described previously [27]. To analyze the expression of adhesion molecules in islets and MSC, both were cultured individually for 72 h and total RNA was extracted using the Qiagen RNeasy Mini kit (Qiagen, San Diego, CA, USA) according to the manufacturer’s instructions. Primers (listed in Additional file 1: Table S1) were designed using Primer3 online software (http://primer3.ut.ee), tested with Primer Biosoft (http://www.premierbiosoft.com), and blasted on BLAST (http://blast.ncbi.nlm.nih.gov/Blast.cgi).

### Cell microencapsulation

The polymer for cell microencapsulation consisted of 5% PEG-8-40 mixed in 1.5% sodium (Na)-Alg, prepared as described previously [30]. Islets alone or with MSC were centrifuged at room temperature (320 × *g* for 2 min) and the pellet was gently mixed with Na-Alg/PEG-8-40 solution (ratio 10–12 islet cells:1 MSC). Microspheres were generated under sterile conditions using the Encapsulator B-395 Pro (Büchi Labortechnik AG, Flawil, Switzerland): the solution in the gelation bath comprised 10 mM MOPS, 100 mM CaCl_2_, and three equivalents of dithiothreitol. Microsphere formation by ionotropic interaction occurred immediately after extrusion in the gelation bath and was completed by covalent crosslinking during subsequent stirring for 30 min in the gelation bath. Alg-PEG microspheres were collected by filtration and washed twice in physiological saline (NaCl, 0.9%) for 10 min to eliminate remaining dithiothreitol.

### Islet transplantation

Animal research was performed following protocols approved by the Geneva cantonal veterinary authorities (license GE/34/13). Diabetes was induced in C57BL/6 male mice (Janvier, France) by intraperitoneal injection of streptozotocin (Sigma, Buchs, Switzerland) at 220 mg/kg. Diabetes was defined as a blood glucose level > 20 mmol/L. Three days after injection, diabetic mice were transplanted. Animals, anesthetized with isoflurane, were transplanted with 4500–5000 IEQ encapsulated islets, with or without MSC (cell ratio 10–12:1) representing a volume of 700 μl of microcapsules, into the peritoneum through a small incision. The peritoneal cavity was used as the site for implantation of capsules, since it has the capacity to receive the high volume of capsules needed to reverse diabetes. Blood glucose was measured 48 h after transplantation and thereafter twice weekly. Islet graft failure was concluded when the glucose level was > 20 mmol/L for three consecutive measurements.

Pseudoislets (with or without MSC) obtained after the in vitro formation were transplanted under the kidney capsule. Because the purpose of this experiment was to analyze the morphology of pseudoislets alone or with MSC in vivo, nonencapsulated pseudoislets were transplanted in nondiabetic immunodeficient SCID mice under the kidney capsule, which allowed histological analysis to be performed. Kidneys were retrieved for histological analyses of the graft. Mouse kidneys were collected at day 15 after transplantation.

### Experimental design of insulin secretion assays and transplantation

For insulin secretion assays using islets or islets with blocking antibodies, comparisons were made for islets alone, islets/MSC in contact, and islets/MSC without contact.

For insulin secretion assays using pseudoislets, comparisons were made for pseudoislets alone, pseudoislets/MSC, and pseudoislets/MSC conditioned medium.

For transplantation of microcapsules in mice, comparisons were made for microcapsules containing islets alone and islets/MSC.

In each experimental setting, islets and MSC derived from two distinct donors (allogeneic) were used.

### Intraperitoneal glucose tolerance test

Fifteen days after transplantation, overnight fasted animals were subjected to the intraperitoneal glucose tolerance test (IPGTT). Glucose (2 g/kg) was injected intraperitoneally and glucose measurements were performed on blood samples collected by tail excision at 0, 5, 15, 30, 60, and 120 min.

### Histological analyses

Pseudoislets after 6 days culture were formalin fixed and paraffin embedded using Histogel (Thermo Scientific, Waltham, MA, USA) following the manufacturer’s recommendations. Four-micrometer sections were treated with 0.01 mol/l citrate for 15 min in a microwave to unmask epitopes. To avoid nonspecific binding, slides were incubated with 0.5% BSA for 30 min at room temperature.

For detection of MSC, sections were stained with mouse anti-human vimentin antibody, diluted 1:50 (Dako, Glostrup, Denmark), and with Alexa Fluor 488 goat anti-mouse antibody (Life Technologies, CA, USA). For detection of islet cells, sections were stained with guinea pig anti-porcine insulin, diluted 1:500 (Dako), donkey anti-guinea pig coumarin AMCA, diluted 1:300 (Jackson Immunoresearch, West Grove, PA, USA), rabbit anti-human glucagon antibody, diluted 1:100 (Merck Millipore, Darmstadt, Germany), and Alexa Fluor 555 donkey anti-rabbit antibody (Life Technologies). Microscopic images were acquired using a fluorescence microscope (Mirax Midi, Zeiss, Germany) and Pannoramic Viewer (3DHISTECH, Hungary), and confocal laser scanning microscopy was performed using LSM700 equipment (Zeiss).

### Statistical analysis

GraphPad Prism software was used. The Mann–Whitney nonparametric test and Wilcoxon signed-rank test were used for in vitro tests, and the nonparametric Kaplan–Meier survival curve and Mantel–Cox tests were used to evaluate the statistical significance for in-vivo graft survival data. For IPGTT the AUC was calculated and values were compared using the parametric *t* test. Differences were considered significant when *p* < 0.05, *p* < 0.01, or *p* < 0.001.

## Results

### MSC improve insulin secretion by human islets in direct cell–cell contact

**Fig. 1:**
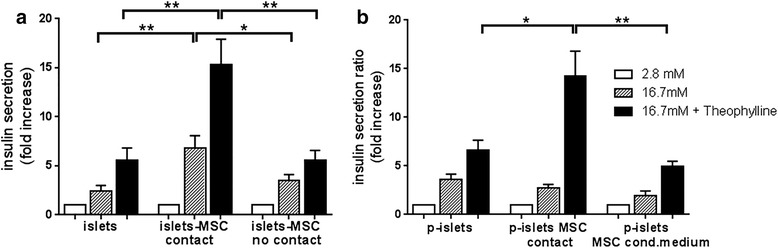
Effect of MSC on insulin secretion by islets. The insulin secretion assay was performed by incubating islets (150 IEQ) or pseudoislets (10^5^ islet cells) at basal glucose (2.8 mM, white bars), high glucose concentration (16.7 mM, striped bars), and high glucose plus theophylline (16.7 and 5 mM, respectively, black bars) for 1 h. **a** Islets alone (islets), together with 15 × 10^3^ MSC (ratio 1 IEQ:100 MSC) in direct cell–cell contact (islets-MSC contact), and islets–MSC without cell–cell contact separated in a transwell chamber (islets-MSC no contact) after 3-day culture. **b** Pseudoislets (p-islets), pseudoislets cultured in direct cell–cell contact with 3 × 10^4^ MSC (ratio 3:1) (p-islets MSC contact), and pseudoislets cultured in MSC-conditioned medium (p-islets MSC cond.medium) after 6-day culture. Values are normalized to the total amount of insulin, and insulin secretion expressed as fold increase where the basal level is set at 1. Data presented as arithmetic mean ± SEM of four or five independent islet donors respectively, and for each donor the assays were performed in triplicate. Mann–Whitney test: *p < 0.05, **p < 0.01

After 3 days of culture, islets alone (islets), islets in direct contact with MSC (islets–MSC contact), and islets and MSC without cell–cell contact (islets–MSC no contact) were subjected to the glucose-stimulated insulin secretion assay. We observed a significantly higher insulin release for islets cultured in contact with MSC than for islets cultured alone (*p* < 0.01) and islets cultured with MSC without cell–cell contact (*p* < 0.05) (Fig. 1a). After maximal stimulation with theophylline, islets in contact with MSC showed a significantly larger increase in insulin secretion than islets alone or islets cultured with MSC without contact (*p* < 0.01). These results show that insulin secretion by human islets is significantly enhanced upon culture with MSC in direct cell–cell contact.

This result prompted us to investigate whether increasing the contact opportunity between MSC and islet cells further increases insulin secretion. Therefore, we cultured dissociated islet cells in hanging drops for 6 days to induce formation of clusters of reaggregated cells, called pseudoislets, without or with MSC. As control, islet cells were cultured in hanging drops in MSC-conditioned medium. All conditions showed similar insulin secretion after high glucose stimulation. However, upon maximal stimulation with theophylline, pseudoislets containing MSC revealed a significantly higher insulin secretion than pseudoislets without MSC (*p* < 0.05) or pseudoislets cultured in MSC-conditioned medium (*p* < 0.01) (Fig. 1b): this indicates a beneficial effect of direct cell–cell contact between MSC and islet cells within pseudoislets. Altogether, these results show that insulin secretion is significantly higher when MSC are in direct cell–cell contact with islets or islet cells in pseudoislets.

### MSC serve as a stromal structure for islets

**Fig. 2:**
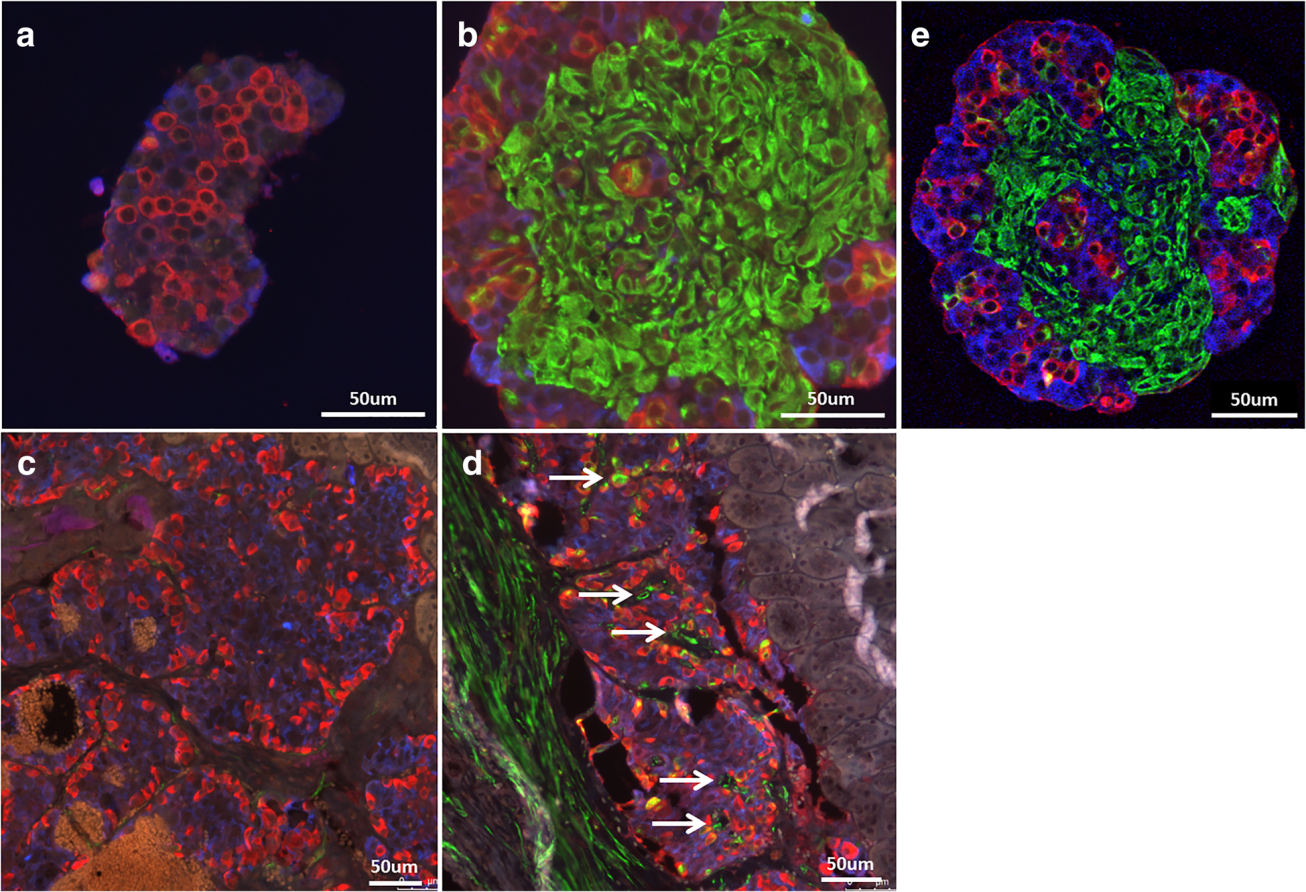
Alpha and beta cell arrangement in cultured pseudoislets and after transplantation in SCID mice. Islets were dissociated by enzymatic digestion, and 10^4^ single cells were cultured in hanging drops for 6 days prior to in vitro immunofluorescence studies, and for 8 days prior to transplantation under the kidney capsule of SCID mice. Beta cells were immunostained for insulin (blue), alpha cells for glucagon (red), and MSC for vimentin (green). Representative images of three different experiments. **a**, **b** Pseudoislets after in vitro formation: (**a**) islet cells alone, (**b**) islet cells and MSC. **c**, **d** Pseudoislets 15 days after transplantation: (**c**) pseudoislets composed of islet cells alone, (**d**) pseudoislets composed of islet cells and MSC. White arrows indicate vimentin-positive cells within the islet graft located outside of the alpha and beta cell arrangements (islet substructures). **e** Right panel: in vitro formed islets and MSC at day 6, confocal laser scanning microscopy

The architecture of native human islets comprises substructures, where centrally located beta cells are surrounded by alpha cells. Histological analysis of in vitro formed pseudoislets showed that alpha and beta cells are oppositely arranged compared to native islets. However, according to the literature the native islet architecture reappears in pseudoislets after transplantation in mice [25]. These data prompted us to assess whether MSC interfere with the structural organization of islet cells in pseudoislets during in vitro culture, and after transplantation in mice. Pseudoislets composed of islet cells formed islet cell clusters composed of centrally located alpha cells, surrounded by a layer of beta cells (Fig. 2a). Pseudoislets containing MSC showed a similar organization of beta cells surrounding alpha cells, and presented single MSC intermingled within alpha and beta cells. Areas, built exclusively of MSC, fit tightly to areas of islet cell clusters (Fig. 2b). Images acquired by confocal laser scanning microscopy revealed a predominant localization of beta cells beside MSC (Fig. 2e).

To analyze the effect of MSC on the architecture of pseudoislets in vivo*,* pseudoislets comprised of islet cells alone or with MSC were transplanted under the kidney capsule of nondiabetic SCID mice and analyzed 15 days later. As shown, pseudoislets without MSC (Fig. 2c) and with MSC (Fig. 2d) reorganized in centrally located beta cells surrounded by alpha cells. MSC in the pseudoislet grafts mostly localized between the substructures of the pseudoislets. In addition, MSC localize around the pseudoislet graft (Fig. 2d, arrows). These results demonstrated that MSC interact with islet cells in vitro and in vivo, further suggesting that MSC serve as a supportive stromal structure for islets.

### Inhibition of N-cadherin decreases the enhanced insulin secretion

**Fig. 3:**
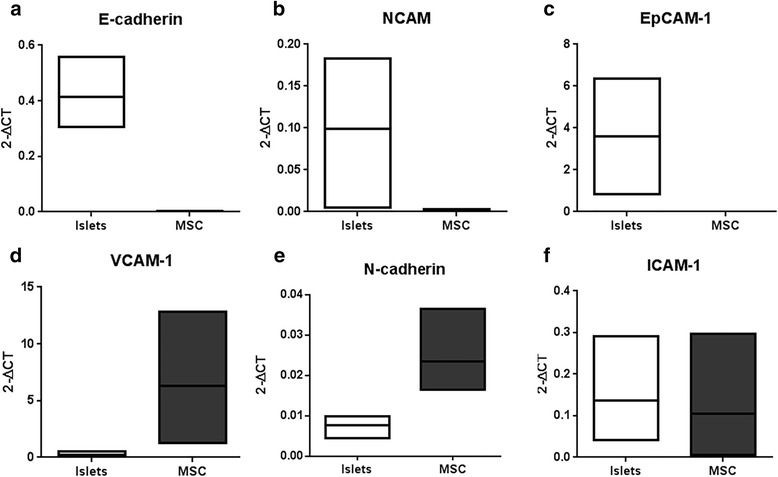
Gene expression of adhesion molecules in islets and MSC. Quantitative RT-PCR was performed to assess adhesion molecule expression and values were normalized to the expression of housekeeping ribosomal protein large P0. Expression of epithelial (E)-cadherin (**a**), neural cell adhesion molecule (NCAM) (**b**), epithelial (Ep)CAM-1 (**c**), vascular (V)CAM-1 (**d**), N-cadherin (**e**), and intercellular (I)CAM-1 (**f**) measured in islets (white boxes) and MSC (black boxes) after a 3-day culture period. Data from three islet donors presented as floating bars (minimum to maximum) with the line presenting the mean value

To investigate whether adhesion molecules are involved in the enhanced insulin secretion, we analyzed separately in islets and MSC the expression of E-cadherin, NCAM, EpCAM-1, VCAM-1, N-cadherin, and ICAM-1. E-cadherin, NCAM, and EpCAM were expressed in islets but not in MSC (Fig. 3a–c). In contrast, VCAM-1 was expressed only in MSC (Fig. 3d). Interestingly, mRNA for N-cadherin and ICAM-1 were expressed both in islets and in MSC (Fig. 3e, f). We therefore selected these adhesion molecules for blocking experiments in the glucose-stimulated insulin assay.

**Fig. 4:**
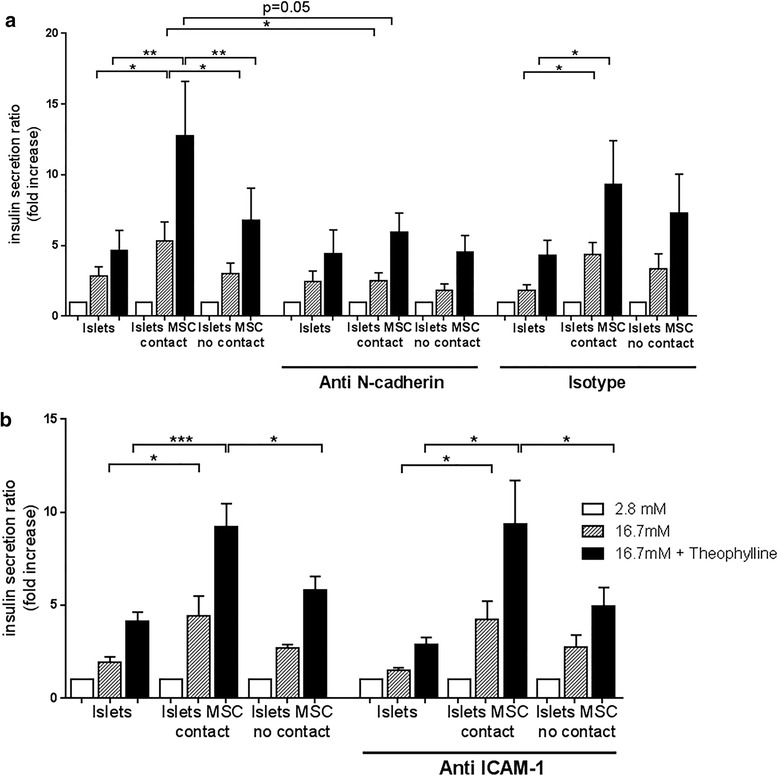
Blocking of N-cadherin but not of ICAM-1 decreased the MSC-enhanced insulin secretion. Antibodies against N-cadherin, ICAM-1, and isotype control were added to the culture of islets (islets), islets and MSC in coculture with cell–cell contact (islets MSC contact) and islets and MSC in culture without contact (islets MSC no contact) for 24 h prior to the insulin secretion assay. Cells were incubated in basal glucose (2.8 mM, white bars), high glucose concentration (16.7 mM, striped bars), and high glucose plus theophylline (16.7 and 5 mM, respectively, black bars) for 1 h. Data are presented as in Fig. 1. **a** Cells cultured alone and with the addition of 25 μg/ml anti-N-cadherin antibody and isotype control. **b** Cells cultured alone and with the addition of 25 μg/ml anti-ICAM-1 antibody. Values of secreted insulin are normalized to the total amount of insulin and expressed as fold increase where the basal level was set at 1. Human islets from four and three different donors used for N-cadherin and ICAM-1 blocking experiments, respectively. For each donor the experiment was performed in triplicate. Each graph represents mean ± SEM. Mann–Whitney test: *p < 0.05, **p < 0.01, ***p < 0.001

Islets with or without MSC were cultured for 24 h in the presence of blocking antibodies against N-cadherin or ICAM-1 and then stimulated to release insulin. Similar to data presented in Fig. 1, islets in contact with MSC showed an increased insulin secretion after high glucose (*p* < 0.05) and theophylline stimulation (*p* < 0.01) when compared to islets alone or islets and MSC cultured separately. Upon culture in the presence of the blocking antibody against N-cadherin, this increase was significantly inhibited, both for glucose stimulation (*p* < 0.05) and for glucose plus theophylline stimulation (*p* = 0.05, Fig. 4a). This inhibition was only observed for cultures in the presence of MSC in direct cell–cell contact with islets. The insulin secretion by islets cultured without MSC or without direct contact with MSC was not affected. The specificity of the observed effect was demonstrated in culture using an isotype control antibody: in this condition, the enhanced insulin release upon glucose and theophylline stimulation was not affected (Fig. 4a). Specific binding of the anti N-cadherin antibody was assessed by FACS and N-cadherin protein expression in islets and MSC was demonstrated by western blot analysis (see Additional file 1: Figure S1). Blocking of ICAM-1 did not inhibit the increased insulin secretion under stimulating conditions, irrespective of the presence of MSC in the culture (Fig. 4b). These results demonstrate that intercellular interactions involving N-cadherin are relevant in enhancing insulin secretion during direct cell–cell contact between islets and MSC.

### Islets microencapsulated with MSC maintain regulated insulin secretion

**Fig. 5:**
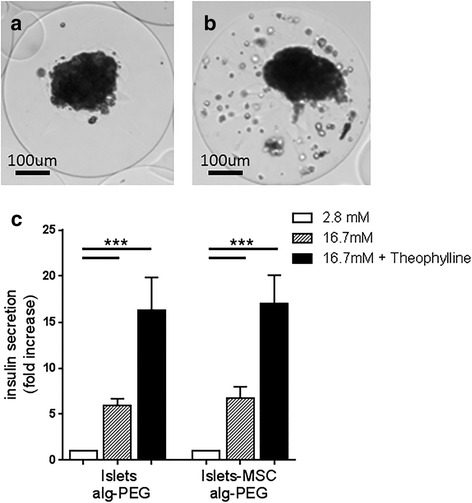
Microencapsulated islets maintain regulated insulin secretion. **a**, **b** Phase contrast images of Alg-PEG microencapsulated islets (10^4^ IEQ/ml Alg-PEG) (**a**) and islets coencapsulated with 8 × 10^5^ MSC (**b**). **c** Insulin secretion by microencapsulated islets (islets Alg-PEG) and islets coencapsulated with MSC (islets-MSC Alg-PEG) at 3 days after encapsulation. Microencapsulated islets (150 IEQ) were incubated at basal glucose (2.8 mM, white bars), high glucose concentration (16.7 mM, striped bars), and high glucose plus theophylline (16.7 and 5 mM, respectively, black bars) for 1 h. Values of secreted insulin are normalized to the total insulin amount and expressed as fold increase where the basal level was set at 1. Data presented as mean value ± SEM of three islet donors, and for each donor the assay was performed in triplicate. Wilcoxon signed-rank test: ***p < 0.001

To evaluate the effect of MSC on the survival and function of microencapsulated islets after transplantation, islets with or without MSC were microencapsulated (Fig. 5a, b). In the microspheres MSC were present in a scattered distribution (Fig. 5b). Three days after microencapsulation, insulin secretion was similar for microencapsulated islets alone and microencapsulated islets with MSC (Fig. 5c). These results show that islets maintained insulin secretion upon glucose stimulation, after microencapsulation. However, early after microencapsulation the enhanced effect on insulin secretion by MSC was not observed. To analyze whether the distribution of MSC inside the microcapsule changed with time, microencapsulated islets and MSC were retrieved 15 days after transplantation. Interestingly, inside the microcapsules MSC localized with islets (see Additional file 1: Figure S2), contrary to MSC in microcapsules before transplantation, where MSC were present in a scattered distribution. This indicates that after transplantation MSC localize to islets in microcapsules and suggests that this interaction fosters glucose-induced insulin secretion.

### MSC prolong survival and function of microencapsulated human islets in diabetic mice

**Fig. 6:**
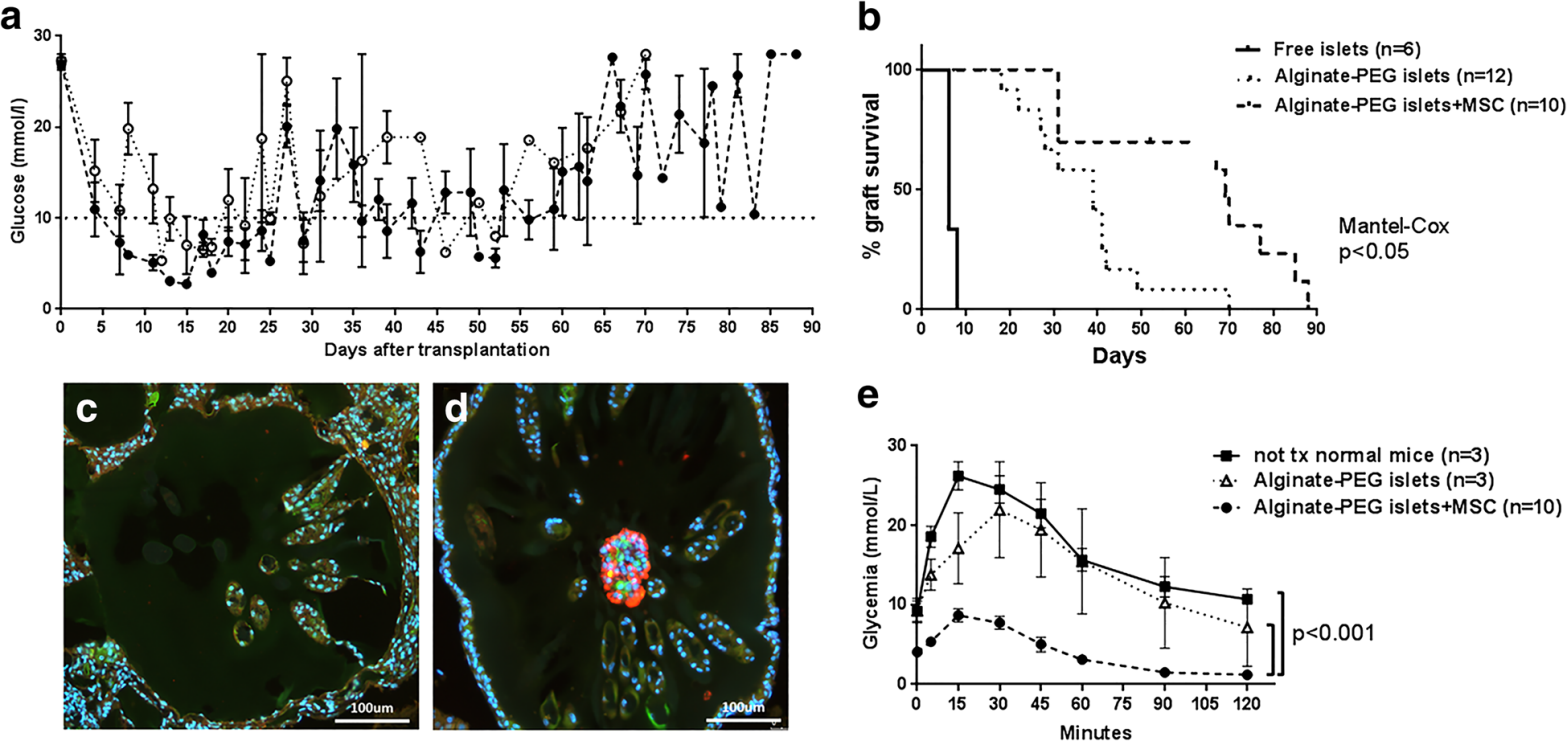
Islet survival and function in diabetic mice. Islets (4.5 × 10^3^–5 × 10^3^ IEQ) were transplanted into streptozotocin-induced diabetic mice. Mice were transplanted with free islets (*n* = 6), with microencapsulated islets (*n* = 12), or with islets coencapsulated with MSC (*n* = 10) at IEQ:MSC ratio 1:80. **a** Glycemia measurements 48 h after transplantation and thereafter twice weekly. Islet graft failure concluded when glucose level was > 20 mmol/L for three consecutive measurements. Dotted line, glucose measurements in 12 mice transplanted with encapsulated islets; dashed line, glucose measurements in 10 mice transplanted with encapsulated islets and MSC. **b** Kaplan-meier curve representing the % of graft survival. Continuous line, mice transplanted with free islets; dotted line, mice transplanted with microencapsulated islets; dashed line, mice trasplanted with islets coencapsulated with MSC. Mantel–Cox test performed. **c**, **d** Beads were retrieved from the peritoneum when mice became diabetic. Sections were immunostained against insulin (red) and vimentin (green) for islets and MSC, respectively. Cell nuclei were stained with Hoechst (blue). **c** Beads recovered from mice transplanted with islets alone. Only empty beads were retrieved. **d** Beads recovered from mice transplanted with islets and MSC together still contained some islets. **e** Intraperitoneal glucose tolerance test performed at day 15 in nontransplanted healthy mice (*n* = 3, continuous line), in diabetic mice transplanted with alginate-PEG islets (*n* = 3, dotted line), and in mice transplanted with alginate-PEG islets + MSC (*n* = 10, dashed line). A single intraperitoneal injection of glucose (2 g/kg) performed at time 0 and glucose monitored at 0, 5, 10, 15, 30, 45, 60, and 120 min thereafter. Values expressed as the mean ± SEM of measurement from at least three mice. Area under the curve is calculated to perform *t*-test statistical analysis

Free islets, microencapsulated islets, and microencapsulated islets with MSC were transplanted into the peritoneum of immunocompetent diabetic C57BL/6 mice. In the immediate post-transplant period, all mice reversed diabetes (Fig. 6a). Mice transplanted with free islets then rapidly lost normoglycemia and became diabetic: the median normoglycemia time was 6 days. Mice transplanted with microencapsulated islets remained normoglycemic until day 18 and then progressively turned diabetic starting at day 20: the median survival time was 39 days. This difference in survival between free and microencapsulated islets was statistically significant (*p* < 0.0001). The period of normoglycemia was significantly longer in mice that received microencapsulated islets with MSC, resulting in a survival period up to 88 days and a median survival of 69 days (*p* < 0.01 compared to microencapsulated islets, Fig. 6b). After graft failure, no insulin-positive cells were found in recovered beads originally containing islets alone (Fig. 6c). However, insulin-positive cells were still present in beads where islets were coencapsulated with MSC (Fig. 6d). These results demonstrate that MSC prolonged human islet graft survival in vivo.

To assess graft function in vivo, the IPGTT was performed at day 15 after transplantation in untreated healthy control mice and diabetic mice transplanted with microencapsulated islets. Blood glucose levels increased 2.6 and 2.4 times over basal glucose levels at time 0, respectively, and recovered to normoglycemia 2 h after injection (ratio 1.2 and 1.3 compared to the basal concentration). Diabetic mice transplanted with microencapsulated islets with MSC manifested lower blood glucose levels than healthy mice at time 0 (4.1 mmol/L compared to 9.1 mmol/L): however, these mice did not show any manifestation of hypoglycemia (weight loss, decreased movements, and erected haircoat). Moreover, after glucose stimulation, these mice showed a lower increase in blood glucose levels (2.0 times compared to time 0) and also presented lower levels after 2 h compared to time 0 (1.2 mmol/L, ratio 0.3). This lower glucose response in comparison with mice transplanted with microencapsulated islets alone reached statistical significance (*p* < 0.001, Fig. 6e). Altogether, these results indicate that human islets coencapsulated with human MSC display a more efficient glucose-induced insulin response in vivo compared to islets alone.

## Discussion

Transplantation of microencapsulated islets without chronic immunosuppression represents a valuable therapeutic option for type 1 diabetic patients, potentially resolving current hurdles associated with clinical transplantation of free islets, such as the adverse side effects of immunosuppressive drugs. However, as for free islets, transplantation of microencapsulated islets does not provide permanent independence from exogenous insulin, and the microencapsulation technology needs further improvements to achieve long-term graft function. Studies in diabetic mice and rats have shown that MSC upon cotransplantation with islets are beneficial for islet function and survival [31]. There are only a few studies in the literature addressing the effect of human MSC on human islets [32–34], which prompted us to assess whether bone marrow-derived human MSC sustain human islet function in vitro and in vivo.

Human islets upon culture with MSC show enhanced insulin secretion in response to glucose and theophylline stimulation (Fig. 1). This potentiating effect of MSC was observed solely when islets and MSC were cocultured in direct cell–cell contact. This observation is supported by findings in rodents, where the contact between MSC and rat islets maintained and increased glucose-induced insulin secretion [35, 36]. Rat islets manifested improved viability when cocultured with MSC at days 7 and 14 [37]; however, in our experimental in vitro conditions, where we performed insulin secretion assays after 3 days of coculture, islet viability was not influenced since total insulin amounts, obtained by extraction with acid ethanol, were comparable in all conditions. Based on the potentiating effect of MSC on insulin secretion, we assessed the direct cell–cell contact between human islet cells and MSC in pseudoislet formation. It has been described that alpha and beta cells in islets reorganize after transplantation of pseudoislets in SCID mice [25]. In the present study, MSC did not alter the arrangement of alpha and beta cells but located between the reorganized substructures, suggesting the integration of MSC as a stromal tissue component in the pseudoislets (Fig. 2).

In mice, optimal regulation of insulin secretion by beta cells is dependent on N-cadherin as demonstrated for a pancreatic epithelium-specific knockout of N-cadherin: N-cadherin controls insulin granule turnover and subsequent insulin secretion [38]. Further, N-cadherin promoted insulin secretion upon stimulation of human beta cells [20]. N-cadherin is expressed in human islets and strongly in human MSC, which led us to investigate the potential role of N-cadherin in the enhanced insulin secretion in coculture with MSC. Blocking N-cadherin interactions for 24 h did not affect insulin expression, since the total amount of insulin was similar in all conditions (data not shown) but abolished the enhanced insulin secretion by islets cocultured with MSC in direct cell–cell contact (Fig. 4a). We concluded that enhanced islet function induced in direct cell–cell contact with MSC requires N-cadherin interactions.

Further, we investigated the effect of human MSC on graft function of microencapsulated human islets in diabetic immunocompetent mice. For transplantation studies, we focused on nondissociated islets since insulin secretion by pseudoislets containing MSC was equivalent compared to nondissociated islets in contact with MSC. This demonstrated that increased contact between islet cells and MSC compared to nondissociated islets did not further increase insulin secretion. The microencapsulation itself prevented acute graft failure due to rejection, from a median survival of 6 days for free islets to a median normoglycemia period of 39 days for microencapsulated islets (Fig. 6b). Coencapsulation of islets and MSC resulted in a further significant prolongation of survival up to 69 days. The IPGTT showed lower blood glucose levels in mice transplanted with islets coencapsulated with MSC (Fig. 6e), which might be attributed to an increased insulin production by islets in the presence of MSC. This phenomenon could also be related to the fact that the presence of MSC in microcapsules protects islets against proinflammatory cytokines. This had been shown in coculture studies, in which human MSC but not human dermal fibroblasts preserved human islets exposed to proinflammatory cytokines [34]. Interestingly, in a pig-to-nonhuman primate xenotransplantation model, the presence of pig MSC in capsules containing pig islets improved oxygenation and neoangiogenesis but only a minor improvement in long-term islet function was observed [39]. Others have shown that the presence of kidney-derived MSC in microencapsulated mouse islets improved graft outcome, even in the absence of MSC-mediated enhancement of revascularization and preservation of islet morphology [31]. Recently, MSC have been described to serve as a “multifunctional islet supportive carrier” for the housing of pancreatic islets in a three-dimensional coculture [40]. Therefore, in order to establish standardized protocols for future application of MSC in clinics, precise mechanisms leading to the supportive effect on islets need to be unraveled.

The strength of this work is based on the finding that molecular interactions of N-cadherin between human islets and MSC are essential for the increased insulin response by islets cocultured with MSC. However, the transplantation setting using encapsulated islets and MSC has some limitations. It remains to be investigated whether N-cadherin interactions are involved in the enhanced and prolonged graft function observed in the coencapsulation setting or whether trophic factors released by MSC are also implicated.

## Conclusions

The present study is the first to show that MSC improved survival and function of microencapsulated human islets transplanted in mice. Altogether, our data suggest that MSC via N-cadherin interactions provide an optimal microenvironment for fine-tuning of insulin secretion and that MSC interactions could be pivotal to support islet graft function in clinical islet transplantation.

## Additional file

Additional file 1:
**Table S1** presenting primers used for adhesion molecule assessment in islets and MSC, **Figure S1** showing FACS and western blot analysis of anti-N-cadherin antibody-binding on MSC, and **Figure S2** showing MSC colocalized with islets in microcapsules 15 days after transplantation. (DOCX 1.34 mb)

## Abbreviations

Alg: Alginate
E-cadherin: Epithelial cadherin
EpCAM-1: Epithelial cell adhesion molecule-1
ICAM-1: Intercellular cell adhesion molecule-1
IEQ: Islet equivalents
IPGTT: Intraperitoneal glucose tolerance test
MSC: Multipotent mesenchymal stromal cells
NCAM: Neural cell adhesion molecule
PEG: Poly(ethylene glycol)
VCAM-1: Vascular cell adhesion molecule-1

## References

[1] Ding Y, Xu D, Feng G, Bushell A, Muschel RJ, Wood KJ (2009). Mesenchymal stem cells prevent the rejection of fully allogenic islet grafts by the immunosuppressive activity of matrix metalloproteinase-2 and -9. Diabetes..

[2] Hematti P, Kim J, Stein AP, Kaufman D (2013). Potential role of mesenchymal stromal cells in pancreatic islet transplantation. Transplant Rev (Orlando).

[3] Perez-Basterrechea M, Obaya AJ, Meana A, Otero J, Esteban MM (2013). Cooperation by fibroblasts and bone marrow-mesenchymal stem cells to improve pancreatic rat-to-mouse islet xenotransplantation. PLoS One..

[4] Ben Nasr M, Vergani A, Avruch J, et al. Co-transplantation of autologous MSCs delays islet allograft rejection and generates a local immunoprivileged site. Acta Diabetol. 2015;52(5):917–27. 10.1007/s00592-015-0735-y PMC4968999 25808641

[5] Borg DJ, Weigelt M, Wilhelm C (2014). Mesenchymal stromal cells improve transplanted islet survival and islet function in a syngeneic mouse model. Diabetologia..

[6] Figliuzzi M, Cornolti R, Perico N (2009). Bone marrow-derived mesenchymal stem cells improve islet graft function in diabetic rats. Transplant Proc..

[7] Hirabaru M, Kuroki T, Adachi T (2015). A method for performing islet transplantation using tissue-engineered sheets of islets and mesenchymal stem cells. Tissue Eng Part C Methods..

[8] Ito T, Itakura S, Todorov I (2010). Mesenchymal stem cell and islet co-transplantation promotes graft revascularization and function. Transplantation..

[9] Rackham CL, Chagastelles PC, Nardi NB, Hauge-Evans AC, Jones PM, King AJ (2011). Co-transplantation of mesenchymal stem cells maintains islet organisation and morphology in mice. Diabetologia..

[10] Giacca M, Zacchigna S (2012). VEGF gene therapy: therapeutic angiogenesis in the clinic and beyond. Gene Ther..

[11] Berman DM, Willman MA, Han D (2010). Mesenchymal stem cells enhance allogeneic islet engraftment in nonhuman primates. Diabetes..

[12] Wu H, Wen D, Mahato RI (2013). Third-party mesenchymal stem cells improved human islet transplantation in a humanized diabetic mouse model. Mol Ther..

[13] Llacua A, de Haan BJ, Smink SA, de Vos P (2016). Extracellular matrix components supporting human islet function in alginate-based immunoprotective microcapsules for treatment of diabetes. J Biomed Mater Res A..

[14] Yang HK, Yoon KH (2015). Current status of encapsulated islet transplantation. J Diabetes Complications..

[15] Meier RP, Navarro-Alvarez N, Morel P, Schuurman HJ, Strom S, Buhler LH (2015). Current status of hepatocyte xenotransplantation. Int J Surg..

[16] Mahou R, Wandrey C (2010). Alginate-poly(ethylene glycol) hybrid microspheres with adjustable physical properties. Macromolecules..

[17] Park KS, Kim YS, Kim JH (2010). Trophic molecules derived from human mesenchymal stem cells enhance survival, function, and angiogenesis of isolated islets after transplantation. Transplantation..

[18] Cirulli V (2015). Cadherins in islet beta-cells: more than meets the eye. Diabetes..

[19] Stewart AF, Hussain MA, Garcia-Ocana A (2015). Human beta-cell proliferation and intracellular signaling: part 3. Diabetes..

[20] Parnaud G, Lavallard V, Bedat B, et al. Cadherin engagement improves insulin secretion of single human beta-cells. Diabetes. 2015;64(3):887–96. 10.2337/db14-0257 25277393

[21] Parnaud G, Gonelle-Gispert C, Morel P (2011). Cadherin engagement protects human beta-cells from apoptosis. Endocrinology..

[22] Brissova M, Fowler MJ, Nicholson WE (2005). Assessment of human pancreatic islet architecture and composition by laser scanning confocal microscopy. J Histochem Cytochem..

[23] Bosco D, Armanet M, Morel P (2010). Unique arrangement of alpha- and beta-cells in human islets of Langerhans. Diabetes..

[24] Maes E, Pipeleers D (1984). Effects of glucose and 3',5'-cyclic adenosine monophosphate upon reaggregation of single pancreatic B-cells. Endocrinology..

[25] Lavallard V, Armanet M, Parnaud G (2016). Cell rearrangement in transplanted human islets. FASEB J..

[26] Ricordi C, Lacy PE, Finke EH, Olack BJ, Scharp DW (1988). Automated method for isolation of human pancreatic islets. Diabetes..

[27] Meier RP, Mahou R, Morel P, et al. Microencapsulated human mesenchymal stem cells decrease liver fibrosis in mice. J Hepatol. 2015;62(3):634–41. 10.1016/j.jhep.2014.10.030 25450712

[28] Lejmi E, Perriraz N, Clement S (2015). Inflammatory chemokines MIP-1delta and MIP-3alpha are involved in the migration of multipotent mesenchymal stromal cells induced by hepatoma cells. Stem Cells Dev..

[29] Meier RP, Seebach JD, Morel P (2014). Survival of free and encapsulated human and rat islet xenografts transplanted into the mouse bone marrow. PLoS One..

[30] Mahou R, Meier RPH, Buhler LH, Wandrey C (2014). Alginate-poly(ethylene glycol) hybrid microspheres for primary cell microencapsulation. Materials..

[31] Kerby A, Jones ES, Jones PM, King AJ (2013). Co-transplantation of islets with mesenchymal stem cells in microcapsules demonstrates graft outcome can be improved in an isolated-graft model of islet transplantation in mice. Cytotherapy..

[32] Fransson M, Brannstrom J, Duprez I (2015). Mesenchymal stromal cells support endothelial cell interactions in an intramuscular islet transplantation model. Regen Med Res..

[33] Johansson U, Rasmusson I, Niclou SP (2008). Formation of composite endothelial cell-mesenchymal stem cell islets: a novel approach to promote islet revascularization. Diabetes..

[34] Yeung TY, Seeberger KL, Kin T (2012). Human mesenchymal stem cells protect human islets from pro-inflammatory cytokines. PLoS One..

[35] Jung EJ, Kim SC, Wee YM (2011). Bone marrow-derived mesenchymal stromal cells support rat pancreatic islet survival and insulin secretory function in vitro. Cytotherapy..

[36] Rackham CL, Dhadda PK, Chagastelles PC (2013). Pre-culturing islets with mesenchymal stromal cells using a direct contact configuration is beneficial for transplantation outcome in diabetic mice. Cytotherapy..

[37] Wang D, Ding X, Xue W (2017). A new scaffold containing small intestinal submucosa and mesenchymal stem cells improves pancreatic islet function and survival in vitro and in vivo. Int J Mol Med..

[38] Johansson JK, Voss U, Kesavan G (2010). N-cadherin is dispensable for pancreas development but required for beta-cell granule turnover. Genesis..

[39] Veriter S, Gianello P, Igarashi Y (2014). Improvement of subcutaneous bioartificial pancreas vascularization and function by coencapsulation of pig islets and mesenchymal stem cells in primates. Cell Transplant..

[40] Borg DJ, Welzel PB, Grimmer M (2016). Macroporous biohybrid cryogels for co-housing pancreatic islets with mesenchymal stromal cells. Acta Biomater..

