# Supplementary material for: Multipotent mesenchymal stromal cells enhance insulin secretion from human islets via N-cadherin interaction and prolong function of transplanted encapsulated islets in mice
Source: Stem Cell Res Ther. 2017 Sep 29;8:199. doi: 10.1186/s13287-017-0646-7 (PMC5622460; doi:10.1186/s13287-017-0646-7)
Supplement: Additional file 1: — Table S1 presenting primers used for adhesion molecule assessment in islets and MSC, Figure S1 showing FACS and western blot analysis of anti-N-cadherin antibody-binding on MSC, and Figure S2 showing MSC colocalized with islets in microcapsules 15 days after transplantation. (DOCX 1.34 mb) [file 13287_2017_646_MOESM1_ESM.docx]

**Additional file**

**Table S1. Primers used in adhesion molecule assessment in islets and MSC**

| **E-cadherin** | Fwd 5’-AGA GAA ACA GGA TGG CTG AAG GTG-3’  Rev 5’-ACT GCA TTC CCG TTG GAT GAC A-3’ |
| --- | --- |
| **NCAM** | Fwd 5’-CCC TCT TCA CCA TCC ATC GA-3’  Rev 5’-TTC ACC AAC TGC TCT CCA CT-3’ |
| **EpCAM-1** | Fwd 5’-CAT GTG CTG GTG TGT GAA CA-3’  Rev 5’-CCA GTA GGT TCT CAC TCG CT-3’ |
| **VCAM-1** | Fwd 5’-GGG AAG ATG GTC GTC ATC CT-3’  Rev 5’-GAT TCT GGG GTG GTC TCG AT-3’ |
| **N-cadherin** | Fwd 5’-GAG CCT GAA GCC AAC CTT AAC TGA-3’  Rev 5’-CTG GCA AGT TGA TTG GAG GGA TGA-3’ |
| **ICAM-1** | Fwd 5’-CGT GGG GAG AAG GAG CTG AA-3’  Rev 5’-CAG TGC GGC ACG AGA AAT TG-3’ |

E-cadherin, epithelial-cadherin; NCAM, neural cell adhesion molecule; EpCAM-1, epithelial cell adhesion molecule; VCAM-1, vascular cell adhesion molecule-1; N-cadherin, neural-cadherin; ICAM-1, intercellular adhesion molecule-1.

**Transplantation of microspheres under the kidney capsule**

Mice were anesthetized with isoflurane and a side incision was performed to approach the kidney. Microspheres (50µl) were transplanted under the kidney capsule using an Abbocath-T 18G catheter (Hospira). Mouse kidneys were collected at day 15 after transplantation and paraffin embedded.

**Histological analyses on microspheres from peritoneum and kidney**

After islet graft failure, mice were euthanized and microspheres were retrieved from the peritoneum, formalin fixed in tubes and then paraffin embedded. Four-µm sections of paraffin embedded microcapsules and kidneys were treated with 0.01mol/l citrate for 15 min in a microwave, to unmask epitopes. To avoid nonspecific binding, slides were incubated with 0.5% BSA for 30 min at room temperature. MSC were stained with mouse anti-human vimentin antibody, diluted 1:50 (Dako, Glostrup, Denmark) and then with Alexa Fluor 488 goat anti-mouse antibody, diluted 1:1,000 (Life Technologies, CA, USA). Beta cells were stained with guinea pig anti-porcine insulin, diluted 1:500 (Dako) and Alexa Fluor 555 goat anti-guinea pig, diluted 1:1,000 (Life Technologies). Microscopic images were acquired using a fluorescence microscope (Leica DM 2000).

**
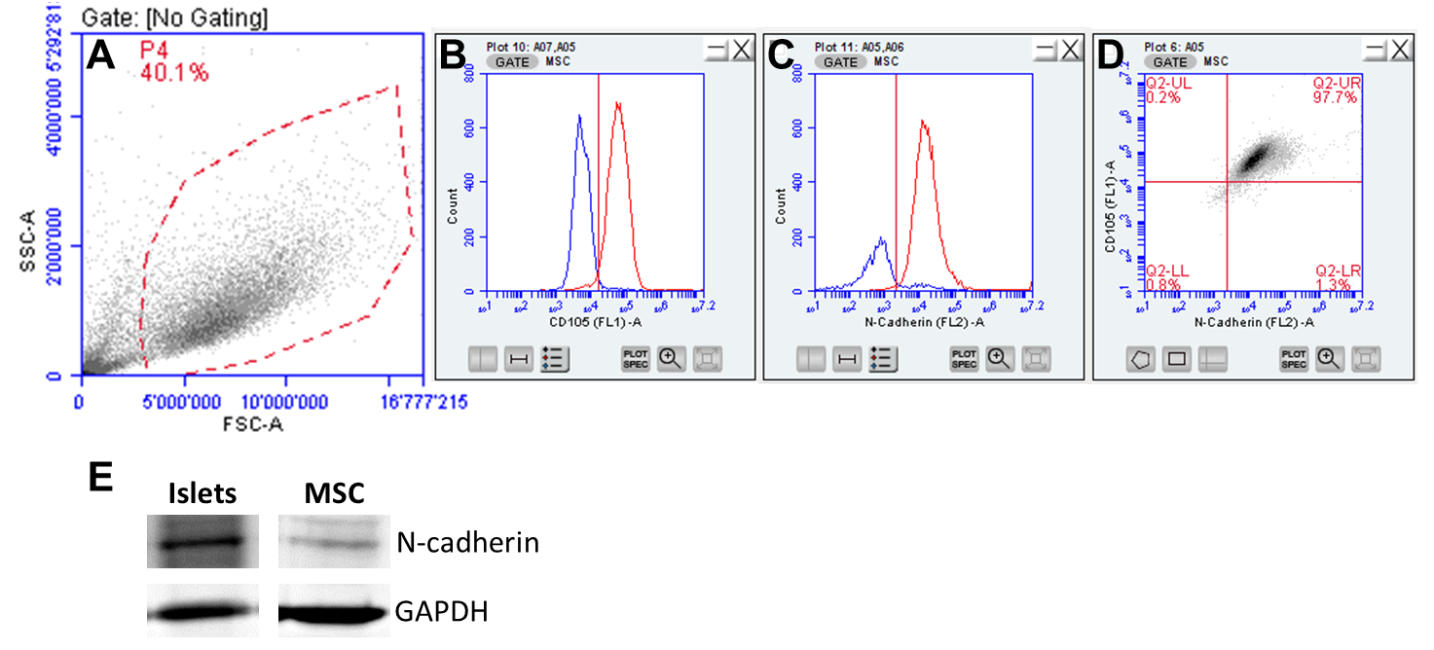
**

**Figure S1. FACS and Western Blot analysis of anti-N-cadherin antibody-binding on MSC. (A)** MSC were gated based on size and cell density. **(B)** MSC were stained by anti-CD105 antibody, a surface antigen expressed on bone marrow stromal cells. Blue line represents unstained MSC and red line represents CD105 positive MSC. **(C)** MSC stained by anti-N-cadherin antibody. Blue line indicates the isotype control and red line indicates N-cadherin positive cells. **(D)** Gated MSC double stained for CD105 and N-cadherin. 97.7% of gated MSC are double positive for CD105 and N-cadherin. **(E)** N-cadherin protein expression was analyzed by western blot, using monoclonal anti-N-cadherin antibody (Sigma). Total protein extracts from human islets (8x10^5^ cells) and human MSC (1x10^5^cells) were loaded on SDS-PAGE.


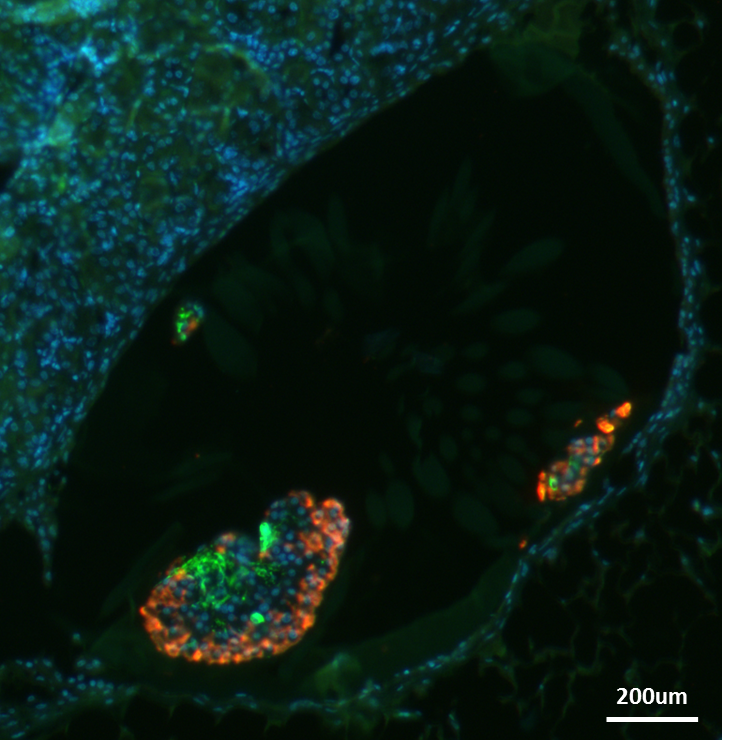


**Figure S2. Co-localization of MSC with islets in microcapsules 15 days after transplantation.** Microspheres transplanted under the kidney capsule were retrieved together with the kidney. Sections were immunostained for insulin (red) and vimentin (green) to stain islets and MSC, respectively. Cell nuclei were stained with Hoechst (blue).
